# Integrating palliative care into multiple myeloma management: Optimizing quality of life across the disease continuum

**DOI:** 10.1007/s00508-024-02447-w

**Published:** 2024-09-27

**Authors:** Lina Rüsing, Christina Brunbauer, Clara Sophie Michel, Claudia Wenzel, Philipp Bauer, Lea Vospernik, Julia Rabensteiner, Feroniki Adamidis, Joachim Baer, Franziska Ecker, Lea Kum, Hermine Agis, Eva Katharina Masel, Maria Theresa Krauth

**Affiliations:** 1 Department of Medicine I, Division Hematology and Hemostaseology, Medical University of Vienna, Waehringer Guertel 18–20, 1090 Vienna, Austria; 2 Department of Medicine I, Division of Palliative Medicine, Medical University of Vienna, Waehringer Guertel 18–20, 1090 Vienna, Austria

**Keywords:** Multiple myeloma, Palliative care, Pain, Symptoms, Early palliative care

## Abstract

**Background:**

Multiple myeloma (MM) poses significant challenges due to its complex symptomatology and evolving treatment landscape. While therapeutic advances have improved survival outcomes, holistic management of MM requires addressing the numerous physical and psychosocial needs of patients. Palliative care (PC) offers a comprehensive approach to symptom management and supportive care on a physical, psychosocial and spiritual level; however, its role in MM remains underexplored.

**Methods:**

This retrospective single-center study examines the outcome of 22 MM patients admitted to the Division of Palliative Medicine at the Medical University of Vienna. We investigated reasons for admission, symptom severity, functional status, length of stay and overall survival.

**Results:**

Most common reasons for palliative care unit (PCU) admission were nutritional problems (82%), fatigue (77%) and pain (68%). Median ECOG score at PCU admission was 3. The timepoint within the timeline of myeloma disease at which hospitalization took place varied greatly. Some patients were hospitalized shortly after diagnosis, other patients after many years of active disease and therapy. Median time from MM initial diagnosis to first PCU stay was 4.3 years (range 0.6–23.8 years). The median length of hospital (PCU) stay was 11 days (range 1–127days) and 45% of patients died during PCU hospitalization. The reduction in symptom burden as a result of the inpatient stay in the PCU is reflected in the PERS^2^ON score, which was measured on the day of admission (median 23 days, range 6–32 days) and on the day of discharge (median 16 days, range 7–20 days).

**Conclusion:**

PC interventions effectively addressed the complex symptom burden experienced by patients with MM. Multidimensional approaches encompassing physical, psychological and social domains proved instrumental in optimizing quality of life. Integrating PC principles into MM management paradigms is essential to prioritize patient-centered care across the disease continuum.

## Introduction

Multiple myeloma (MM) is a paradigmatic example of the evolving landscape of cancer treatment. With advances in therapeutic modalities, including novel targeted agents and immunotherapies, patients diagnosed with MM are now experiencing prolonged survival and improved disease control across multiple lines of treatment [1, 2]. Despite these remarkable advances, the journey of people living with MM remains fraught with challenges, most notably a high symptom burden that encompasses both the physical and psychosocial domains [3].

While the prognosis for MM patients has improved significantly with the advent of multidisciplinary treatment approaches, the complexity of managing this hematologic malignancy goes beyond simple survival metrics. Indeed, the hallmark of MM is not only the response to therapy, but also in the quality of life (QoL) experienced by those living with the disease [4]. This is underlined by the persistent symptomatology that accompanies the disease course, including debilitating bone pain, fatigue, frailty, neuropathy and emotional distress [5–7].

Amidst the clinical focus on disease-modifying interventions, the integral role of palliative care (PC) in optimizing the well-being of MM patients is often underemphasized [8–11]. PC, characterized by its holistic approach to symptom management, including psychosocial as well as spiritual support and advance care planning, is of paramount importance in addressing the multiple needs of persons facing the challenges of MM [12, 13].

By shifting the paradigm from a predominantly disease-centered model to one that prioritizes patient-centered care, PC interventions offer an opportunity to improve QoL, foster resilience, and cultivate a sense of empowerment amidst the complexities of the disease [14, 15].

The aim of this retrospective study was to explore the impact of PC among MM patients, and to delineate the evolving landscape of symptom management and supportive interventions tailored to the unique needs of MM patients. Through an examination of current evidence and emerging trends, we highlight the critical role of early comprehensive PC in improving patient outcomes, mitigating treatment-related toxicities, and fostering resilience in the face of adversity.

By advocating the integration of PC principles into the management of MM, we strive for a future in which the pursuit of optimal QoL is imperative in the continuum of care for individuals affected by this complex hematologic malignancy.

## Patients, material and methods

Data of patients with MM treated at the Division of Palliative Medicine, Medical University of Vienna, between December 2017 and December 2023 were retrospectively reviewed. The Vienna General Hospital comprises about 1700 beds with an integrated Comprehensive Cancer Center and a PC unit comprising 12 beds. The PCU has about 300 admissions per year.

Patient characteristics (age, sex, time since MM diagnosis), ECOG performance status, symptoms at admission, treatment and interventions during hospitalization, and clinical outcomes (OS) were determined. Symptom burden on admission and discharge was assessed using the PERS^2^ON score, a tool developed for PC patient-centered medical history to capture patients’ most important complaints. It rates seven items, i.e., **p**ain, **e**ating (loss of appetite/weight loss), **r**ehabilitation (physical handicap), **s**ocial situation (possibility for home care), **s**uffering (anxiety/burden of disease/depression), **O**2 (dyspnea) and **n**ausea/emesis, on a scale ranging from 0 (absence) to 10 (worst imaginable), resulting in a score ranging from 0 to a maximum of 70 [16]. Due to the small number of cases, we have refrained from a statistical analysis and focus on a descriptive presentation of the results in this paper. The study was conducted in accordance with the Declaration of Helsinki and was approved by the Ethics Committee of the Medical University of Vienna (EK number: 1941/2020).

## Results

The study included 22 patients with MM admitted to the Division of Palliative Medicine. They had the following clinical characteristics: 41% women, 59% men; median age 75 years (range 53–90 years); median ECOG 3 (Table 1).

**Table 1:** Characterization of patients

Age (years, median; range)	74 (53–90)
*Sex*	
Female (*n*, %)	9 (41%)
Male (*n*, %)	13 (59%)
*Clinical symptoms at admission to the PCU*	
Nutritional problems (cachexia ± lack of appetite ± anorexia)	18 (82%)
Fatigue	17 (77%)
Pain	15 (68%)
Cachexia	10 (45%)
Weakness	13 (59%)
Lack of appetite	8 (36%)
Dyspnea	7 (32%)
Nausea	3 (14%)
Ascites	3 (14%)
Polyneuropathy	3 (14%)
Anorexia	3 (14%)
*ECOG (median, range)*	3 (2–4)
*PERS*^*2*^ON score at admission day* (median, range)	23 (6–32)
*PERS*^*2*^ON score at discharge day** (median, range)	16 (7–20)
*Time since MM diagnosis in years (median, range)*	4.3 (0.6–23.8)
*Time since last MM Therapy in days (median, range)*	14 (2–80)
*MM therapy during PCU stay (n, %)*	7 (32%)
*Disease status at PCU admission day*	
Progressive disease (PD)	14 (64%)
Stable disease (SD)	6 (27%)
Complete response (CR)	2 (9%)
*OS from PCU admission (median, range)*	1.4 months (1 day–3.8 years)
*Stay at PCU (days) (median, range)*	11 (1–127)
*Death at PCU (n, %)*	10 (45%)
*Advance care planning (APC) (n, %)*	0

*Data missing for 5 patients, **Data missing for 7 patients

*ECOG* Eastern Cooperative Oncology Group performance status, *PERS*^*2*^*ON score* tool developed for PC patient-centered medical history to capture patients’ most important complaints (see material and methods section), *PCU* Palliative care unit

All patients had received prior MM treatment including anti CD-38Ab (86%), immunomodulatory drugs (95%), autologous stem cell transplant (32%). Median time since last MM treatment was 14 days (range 2–80 days). Seven patients (32%) further received anti MM treatment during their PCU stay, 14 patients (64%) had progressive disease (PD) at PCU admission, 6 patients (27%) had stable disease and 2 patients (9%) were in CR at the time of PCU admission.

The main reasons for admission were nutritional problems (either cachexia, lack of appetite or anorexia) in 82%, fatigue in 77%, uncontrolled MM-related pain in 68%, weakness in 59% and dyspnea in 32%.

The median length of hospital (PCU) stay was 11 days (range, 1–127days) and 45% of the patients died during hospitalization. No patients had advance directives.

**Fig. 1:**
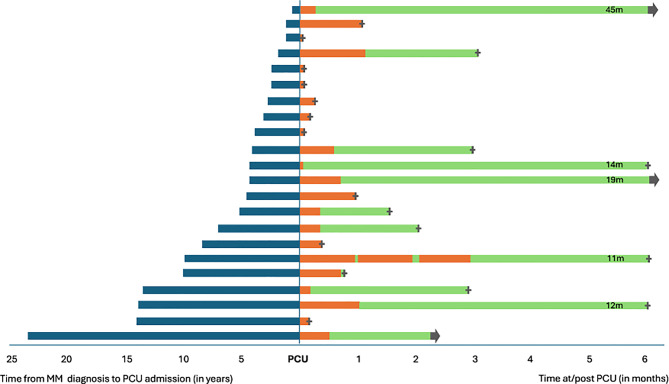
Butterfly plot—Time from initial MM diagnosis to PCU admission (*blue*). Months at PCU (*orange*), months after PCU (*green*)

The timepoint within the timeline of myeloma disease at which PCU hospitalization took place varied greatly. Some patients were hospitalized shortly after diagnosis, other patients after many years of active disease and treatment (Fig. 1). Only 1 patient had more than 1 PCU stay and was admitted 3 times with only 2 and 5 days at home between the PCU stays.

Median time from MM initial diagnosis to first PCU stay was 4.3 years (range 0.6–23.8 years), median length of stay at the PCU was 11 days (range 1–127days). Median survival from PCU admission to death was 1.4 months with a wide range between 1 day and 3.8 years.

**Fig. 2:**
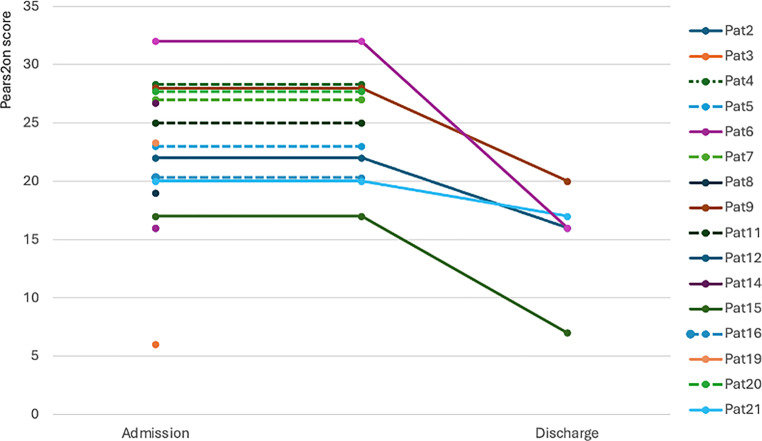
PERS^2^ON score on admission day and on discharge day. *Dashed line* death on the PCU, *continuous line* no death on PCU, *no line* PERS^2^ON score was assessed at admission only, not at discharge

Improvement or stabilization of tumor-related symptoms was achieved in all patients where a PERS^2^ON score at discharge was assessed (Fig. 2). Median PERS^2^ON score at admission was 23 (range 6–32). Median PERS^2^ON score on discharge day was 16 (range 7–20).

## Discussion

In this retrospective study, we analyzed the outcomes of 22 patients diagnosed with MM who were admitted to the PCU for comprehensive symptom management. Here, we present a detailed discussion of the results observed in this cohort.

Admission to PCU took place at various timepoints after MM diagnosis and ranged between a few months to over 20 years after initial diagnosis of MM.

The most common clinical symptom on admission to the PCU was nutritional problems (cachexia; loss of appetite; anorexia), followed by fatigue and pain. In a study by Pallotti et al. of 43 patients, the most common reason for PCU consultation was pain [17]; however, in their study, PCU consultation was mainly outpatient (74%), whereas in our study, we included only inpatient PCU patients. But it is important to highlight outpatient PC as well as it plays a crucial role in the continuum of support for MM patients. PC provides ongoing symptom management, psychological support, and coordination of care, enabling patients to maintain their quality of life at home. Integrating outpatient PC early in the treatment process ensures that patients receive comprehensive and consistent care, addressing a wide range of needs and potentially reducing the frequency of hospital admissions.

Fatigue emerged as a prominent symptom among the patients included in our study. Despite advances in the treatment of MM, fatigue remains a pervasive issue, significantly impacting patients’ quality of life [18]. Through tailored interventions focusing on energy conservation, activity pacing, and pharmacological management, we observed significant improvements in fatigue levels in the majority of patients. Multidisciplinary approaches, including physical and psychosocial therapy, have been instrumental in addressing the multifaceted nature of fatigue in MM [19, 20].

Nutritional problems represent an important aspect of supportive care in MM patients, with anorexia, cachexia and malnutrition being significant barriers to treatment tolerance and disease management. Through a comprehensive nutritional assessment and implementation of dietary interventions tailored to individual patient needs, we observed improvements in nutritional status and appetite in a subset of patients. Nutritional support, including oral supplements and enteral nutrition, played a pivotal role in improving malnutrition and enhancing treatment adherence [21, 22].

Pain, a hallmark symptom of MM, has a profound impact on patients’ well-being and functional status. Our findings highlighted the effectiveness of multimodal analgesic strategies, including pharmacological management, physiotherapeutic interventions, and multimodal therapies in achieving optimal pain control and improving QoL [23].

**Fig. 3:**
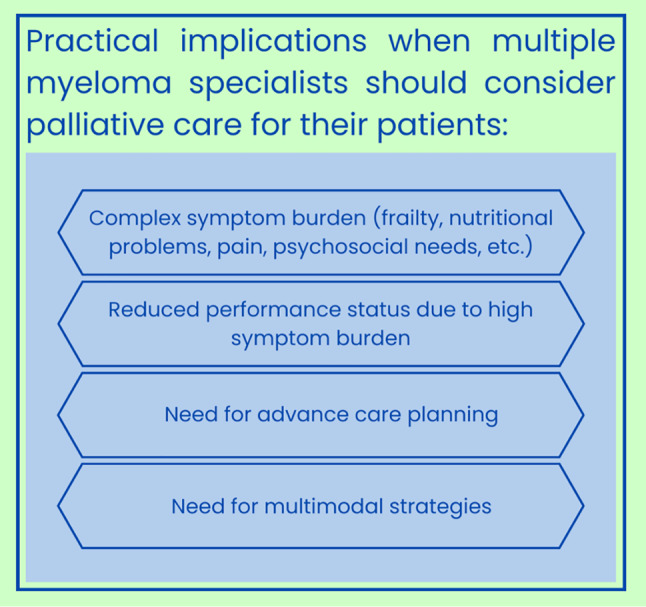
Practical implications—when to consider PC specialist

Our results show that admissions to the palliative care unit (PCU) were often necessitated by both tumor-related symptoms and treatment complications; however, it is often difficult to distinguish between treatment-related and disease-related symptoms, such as fatigue, weakness, and nutritional problems, as these symptoms are often non-specific. This highlights the dual role of PC in managing both the direct effects of the malignancy and the side effects associated with its treatment. By addressing these complex and intertwined issues, PC not only alleviates physical suffering but also may improve overall quality of life, highlighting its indispensable role in comprehensive cancer care. Integrating PC early into the treatment process can therefore provide holistic support and address a wide range of patient needs. Fig. 3 highlights practical implications, when MM-specialist should consider palliative care for their patients.

Patient survival after discharge from the PCU varied, with 45% of patients dying in the hospital and the remaining 55% having a median overall survival of 1.4 months, with a wide range from 1 day to over 3 years. These data show that even after a stay in the PCU, MM patients can have a long life ahead of them. This underlines the benefit of early integration for successful symptom relief and comprehensive, individualized treatment.

The fact that none of the patients had an advance care document highlights the importance of further measures to promote the concept of advance care planning (ACP). This is an essential approach to ensure that a person’s healthcare preferences and decisions are known and respected. Given the unpredictable nature of MM and its potential for progression, discussing and documenting patients’ preferences regarding their future healthcare decisions is of paramount importance for this patient cohort. The ACP involves open dialogue between patients, their families, and healthcare providers to clarify goals of care, explore treatment options, and address QoL concerns. These discussions may include preferences for pain management, resuscitation, hospice care, and end-of-life wishes. By engaging in ACP, MM patients and their environment can navigate the complexities of the disease with greater understanding and ensure that care is consistent with their values and preferences [24, 25].

## Strength and weaknesses of this study

The retrospective nature of this study warrants cautious interpretation of the results. Retrospective studies rely on existing data, which may be subject to bias, incomplete records or inaccuracies. In addition, the focus on PC consultations within a single center may limit the generalizability of the results to broader populations or healthcare settings. Therefore, although the findings provide valuable insights, they should be carefully contextualized within the specific scope of this study design.

## Conclusion

Overall, our findings highlight the integral role of PC in addressing the complex symptom burden experienced by patients with MM. By adopting a multidimensional approach that encompasses physical, psychological, and social domains, PC interventions have the potential to optimize QoL, enhance resilience, and empower individuals to cope with the challenges posed by MM.

By continuing to advocate for the integration of PC principles into MM management paradigms, we aim to prioritize patient-centered care and holistic well-being in the MM treatment continuum.

## Abbreviations

ACP: Advance care planning
MM: Multiple myeloma
PC: Palliative care
PCU: Palliative care unit
QoL: Quality of life
